# Person-centered approach to suicide ideation in posttraumatic stress disorder in veterans: a latent profile analysis

**DOI:** 10.1192/j.eurpsy.2024.1376

**Published:** 2024-08-27

**Authors:** M. Vilibić, S. Nadalin, B. Aukst Margetić, Ž. Šurina Osmak, V. Peitl, D. Karlović

**Affiliations:** ^1^Department of Psychiatry, University Hospital Centre Sestre milosrdnice; ^2^School of Medicine, Catholic University of Croatia, Zagreb; ^3^Department of Psychiatry, General Hospital “Dr Josip Benčević”, Slavonski Brod; ^4^Department of Health, Ministry of Defence of the Republic Croatia, Zagreb, Croatia

## Abstract

**Introduction:**

While most research on suicidal ideation (SI) in veterans adopts a variable-oriented perspective, this approach often fails to capture the complex interplay of symptoms and comorbid disorders. We hypothesised that a person-centred approach can identify distinct subpopulations of veterans with varying profiles of SI, PTSD symptoms, depression, and agitation.

**Objectives:**

To examine whether distinct subpopulations of veterans exists, characterized by different profiles of PTSD severity, depression and agitation, and intensity of SI.

**Methods:**

We conducted a cross-sectional study in one big University Hospital Centre in Croatia on the sample of men, war veterans aged 30-65 years, undergoing treatment for chronic PTSD. Latent profiles indicators included the Clinician-Administered PTSD Scale (CAPS), Beck Scale for Suicide Ideation (SSI), Hamilton Depression Rating Scale-17 (HDRS-17) and Corrigan Agitated Behaviour Scale (CABS).

**Results:**

We included 203 male participants with a median age of 47 (IQR 43-45) years. The optimal model, allowing variances of indicators to vary between profiles while constraining covariances to zero, yielded five distinct latent profiles. Notably, the highest SI was found in a subpopulation with elevated CABS scores, but moderate PTSD and depression symptoms (13% of participants). Next in SI intensity were 11% of veterans with severe symptoms across all assessed disorders. Next in SI severity were 21% of veterans with low levels of agitation but high levels of depression. The last two profiles, one with mild symptoms of all assessed disorders (43%) and the other with high agitation (12%), have low SI severity.

**Conclusions:**

Our findings affirm the utility of a person-centred approach in identifying nuanced subpopulations of veterans with diverse symptom profiles related to SI. This stratification can inform targeted interventions, thereby enhancing the efficacy of suicide prevention strategies.

**Disclosure of Interest:**

None Declared

